# Prevalence and Predictors of COVID-19 Long-Term Symptoms: A Cohort Study from the Amazon Basin

**DOI:** 10.4269/ajtmh.22-0362

**Published:** 2023-06-26

**Authors:** Kletey M. Silva, Dhayn C. A. Freitas, Sabrina S. Medeiros, Laís V. A. Miranda, Jessica B. M. Carmo, Roberta G. Silva, Luana L. Becker, Emanuel S. Abreu, Leonardo Buranello, Maria S. M. Souza, Wilson Nadruz, Miguel M. Fernandes-Silva, James H. Maguire, Cristina Toledo-Cornell, Odilson M. Silvestre

**Affiliations:** ^1^ Federal University of Acre, Rio Branco, Brazil;; ^2^ University of Campinas, Campinas, Brazil;; ^3^ Federal University of Parana, Curitiba, Brazil;; ^4^ Division of Infectious Disease, Brigham and Women’s Hospital, Harvard Medical School, Boston, Massachusetts;; ^5^ Department of Medicine, Brigham and Women’s Hospital, Harvard Medical School, Boston, Massachusetts

## Abstract

It remains unclear whether a previous history of tropical infectious diseases and a second SARS-COV-2 infection may influence the likelihood of later symptoms. In this prospective cohort study, individuals infected with SARS-CoV-2 were followed up by telephone shortly after diagnosis of COVID-19 and again 12 months later. Poisson regression was used to identify the predictors of the highest number of symptoms in the post-COVID-19 syndrome. A total of 1,371 patients with COVID-19, with a mean age of 39.7 ± 11.7 years and 50% female, were followed for 12 months. Reinfection was found in 32 (2.3%) participants, and 806 (58.8%) individuals reported a previous history of dengue, malaria, Zika, chikungunya, leprosy, and visceral leishmaniasis. Eight hundred seventy-seven (63.9%) participants reported late symptoms related to COVID-19. After adjusting for multiple factors, female sex, non-White race, number of acute-phase symptoms, body mass index, and reinfection were independent predictors of higher number of symptoms in post-COVID-19 syndrome. Female sex, non-White race, number of acute-phase symptoms, body mass index, and reinfection, but not previous endemic tropical diseases, were associated with long-term symptoms.

## INTRODUCTION

Although more than 500 million persons have been infected with SARS-CoV-2 worldwide,^1^ there are gaps in our understanding of long-term outcomes, especially after reinfection. Common symptoms in the symptomatic acute phase of COVID-19 include fever, cough, fatigue, myalgia, and headache and last up to 28 days.^2^ However, patients with even mild symptoms in the acute phase may have symptoms persisting for a prolonged period of time or develop new symptoms after the initial phase of the infection.^3^ Several long-term follow-up studies described late symptoms of COVID-19 ranging in 18% to 89% of the patients, commonly, fatigue, sleep disorder, dyspnea, cough, arthralgia, and chest pain.^4^^,^^5^ However, the lack of a uniform definition of post-COVID-19 syndrome has made it difficult to determine the incidence or identify predictors of post-COVID-19 syndrome. Moreover, it is unclear whether a previous contact with tropical infectious diseases and/or SARS-COV-2 reinfection increase the probability of late symptoms.

In the Amazon Basin, tropical diseases such as dengue, malaria, Zika, chikungunya, leprosy, and visceral leishmaniasis are endemic and may have affected more than half of population.^6^ A previous analysis from the Amazon Basin found a higher prevalence of COVID-19 acute symptoms among those with previous dengue infection.^7^ SARS-COV-2 reinfection most commonly occurs when the host comes into contact with a new variant of the virus, and symptoms may be worse the second time around.^8^

We hypothesized that late symptoms are common in this area with a high prevalence of tropical disease; moreover, these infections and the occurrence of a second SARS-CoV-2 infection increases the probability of late symptoms of SARS-CoV2.

## MATERIALS AND METHODS

### Study design and sample.

In this prospective cohort study, we studied 1,371 individuals infected with laboratory-confirmed SARS-CoV-2 in Rio Branco, Acre, a municipality in the Brazilian Amazon Basin. These were among the 9,878 cases of COVID-19 confirmed between March 17 and August 26, 2020, whose information was provided to us by the Secretary of Health of Acre State and the Health Secretary of the city of Rio Branco. We attempted to contact subjects by telephone shortly after their diagnosis of COVID-19 and again approximately 8 to 14 months later. As shown in Supplemental Figure 1, the final sample was composed of two independent groups. The first group consisted of 1,339 individuals who had one SARS-CoV-2 infection (primary infection), and the second group consisted of 32 individuals who had two SARS-CoV-2 infections (reinfection). The current study sample was composed from a previous published cohort study and no sample size calculation was made for this subanalysis.^7^

Data were collected on sociodemographic characteristics, clinical status before SARS-CoV-2 infection, and clinical details about the acute phase of the disease. Baseline characteristics included weight, height, smoking status, sedentary lifestyle, alcohol consumption, and relevant comorbidities; a history of previous endemic infectious diseases (dengue, malaria, Zika, chikungunya, leprosy, and visceral leishmaniasis) was self-reported; details about hospitalization for COVID-19 and symptoms present in the acute phase of the infection were recorded, and in a 12-month follow-up questionnaire, participants were asked about persistent symptoms, new symptoms, and details concerning reinfection.

We defined long-term post-COVID-19 as self-reported symptoms that persisted for 12 months after a confirmed diagnosis of SARS-COV-2 without an alternative explanation.^9^ SARS-COV-2 reinfection was defined following the CDC criteria—specifically, a positive reverse-transcriptase (RT)-PCR transcription assay at two distinct time points at least 90 days apart.^10^ Participants who reported a second infection at the 12-month follow-up were asked to send a positive RT-PCR test result via WhatsApp. The study was approved by the Research Ethics Committee of the Federal University of Acre, Brazil, and all participants signed the free and informed consent term.

### Statistical analysis.

Categorical and continuous variables with normal distribution were expressed as absolute frequencies and percentages (%) and mean ± SD, respectively. Continuous variables without normal distribution were expressed as median with interquartile range. Comparisons between the primary infection and reinfection groups were evaluated using χ^2^, Fisher’s exact, and Student’s *t* tests for variables with normal distribution and Mann–Whitney test for variables without normal distribution. To identify predictors of post-Covid-19 syndrome, we performed a Poisson regression model because the endpoint was the number of late symptoms for each participant. First, Poisson bivariate regression was performed to identify predictors of post-COVID-19 syndrome, and variables with *P* values < 0.05 were included in an adjusted multivariate Poisson regression to identify independent predictors. To evaluate the potential effects of selective attrition, we performed a sensitivity analysis using inverse probability weighting (IPW). We compared the baseline characteristics of individuals of the original cohort between those who attended versus those who did not attend the 12-month phone visit. We used the baseline characteristics with complete data to ascribe weights as the inverse probability of attending the 12-month visit. This analysis was presented in the Supplemental Material. No adjustment for multiple tests were performed. Statistical analyses were performed with Stata 14.1 (Stata Corp., College Station, TX, USA). Values of *P* < 0.05 were considered statistically significant.

## RESULTS

The mean age of the 1,371 participants was 39.7 ± 11.7 years; 50.7% were female, the median number of symptoms in the acute phase was 9, and 7% of individuals were hospitalized (Table 1). A total of 32 (2.3%) participants met criteria for reinfection with SARS-CoV-2. History of previous dengue, malaria, Zika, chikungunya, leprosy, and visceral leishmaniasis was found in 806 (58.8%). Participants with COVID-19 reinfection did not show significant differences in sociodemographic and clinical variables compared with those with only primary infection.

**Table 1:** Baseline sociodemographic and clinical characteristics of patients with primary infection and reinfection

Characteristics	Total (*N* = 1,371)	Primary infection (*N* = 1,339)	Reinfection (*N* = 32)	*P* value
Age (years), mean ± SD	39.7 ± 11.7	39.7 ± 11.6	39.7 ± 11.2	0.98
Female, *n* (%)	695 (50.7)	680 (50.7)	15 (48.8)	0.66
Race, *n* (%)				
White	251 (18.4)	247 (18.5)	4 (12.5)	0.54
Brown	953 (69.8)	931 (69.8)	22 (68.8)	
Black	124 (9.1)	119 (8.9)	5 (15.6)	
Other	38 (2.8)	37 (2.8)	1 (3.1)	
Smoking, *n* (%)	70 (5.1)	70 (5.2)	0 (0.0)	0.40
Alcohol, *n* (%)	211 (15.4)	207 (15.4)	4 (12.5)	0.80
Sedentary lifestyle, *n* (%)	706 (51.5)	686 (51.2)	20 (62.5)	0.20
Obesity, *n* (%)	446 (32.5)	436 (32.5)	10 (31.2)	0.87
Comorbidities, *n* (%)				
Hypertension	255 (18.6)	248 (18.5)	7 (21.8)	0.63
Chronic heart diseases	44 (3.2)	42 (3.1)	2 (6.2)	0.27
Diabetes mellitus	75 (5.4)	73 (5.4)	2 (6.2)	0.69
Chronic lung disease	57 (4.1)	56 (4.1)	1 (3.1)	1.0
Chronic kidney disease	22 (1.6)	22 (1.6)	0 (0.0)	1.0
Hospitalization, *n* (%)	97 (7.0)	96 (7.1)	1 (3.1)	0.72
No. of symptoms (acute phase), median (IQR)	9 (6–12)	9 (7–12)	8 (4.5–10.5)	0.09
Time since primary infection (months), median (IQR)	11.9 (11.5–12.4)	11.9 (11.5–12.4)	11.8 (11.5–12.2)	0.21

IQR = interquartile range.

**Figure 1.**
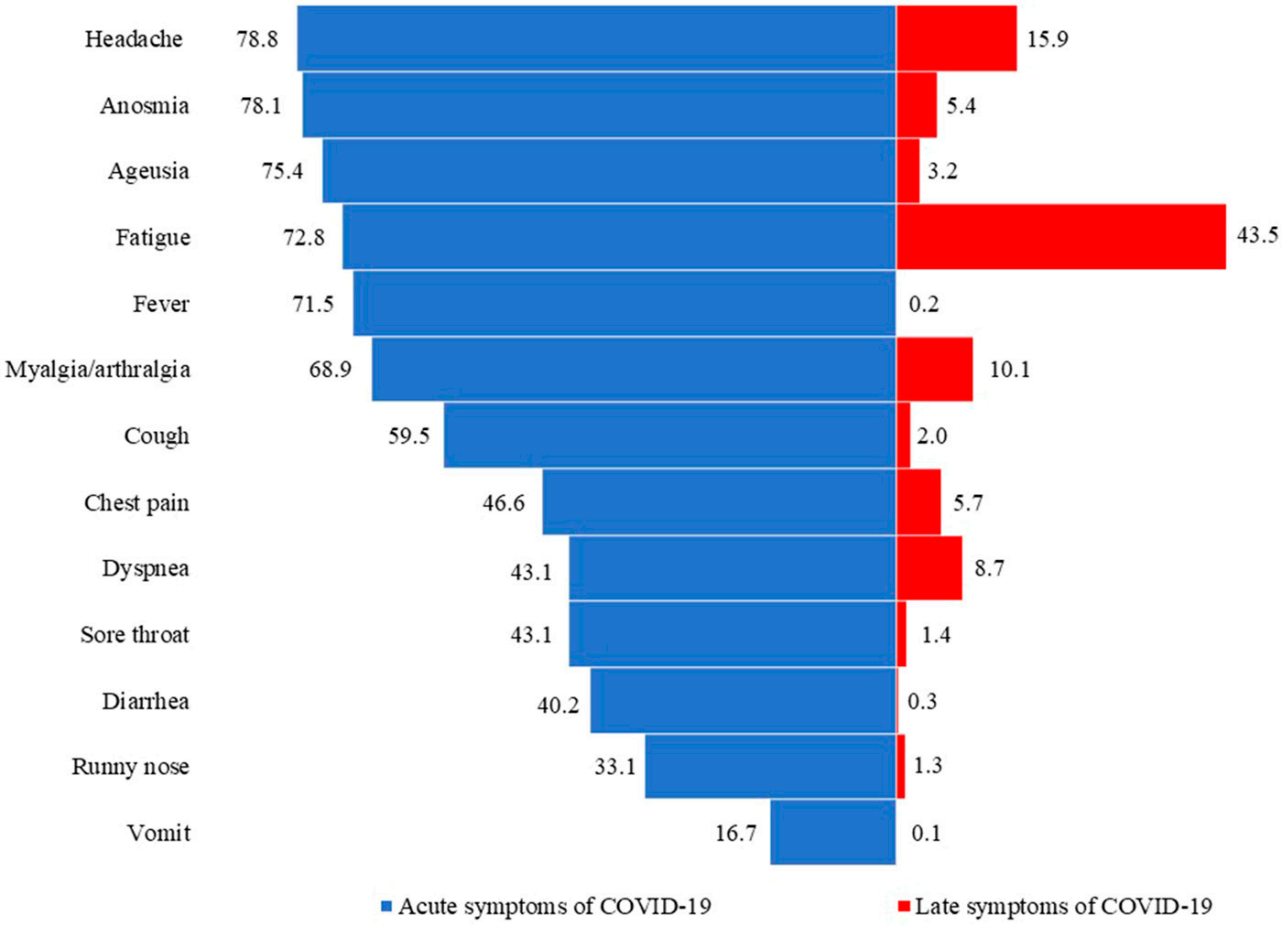
Symptoms related to COVID-19. The percentage of symptoms during the acute phase (blue) and late symptoms after 12 months (red), *N* = 1,371.

The most frequent symptoms during the acute phase of COVID-19 were headache, anosmia, ageusia, fatigue, fever, myalgia/arthralgia, and cough. Twelve months after the primary infection, 63.9% of subjects reported at least one persistent symptom related to SARS-CoV-2 infection. The most frequent late symptoms reported by the participants were fatigue, headache, myalgia/arthralgia, dyspnea, chest pain, and anosmia (Figure 1).

Compared with primary infection subjects, those who experienced reinfection had higher incidence of late symptoms (63.4% and 87.5%, *P* = 0.005, respectively), the most common symptoms being fatigue, headache, dyspnea, myalgia/arthralgia, and cough. Those who had reinfection had more frequent cough but less frequent back pain, anosmia, and ageusia (Supplemental Table 1) compared with primary infection patients. The interval between reinfection and the 12-month follow-up was a median of 2.3 (1.1–3.4) months.

Through bivariate regression, female sex, non-White race, number of comorbidities, number of symptoms in the acute phase, body mass index, sedentary lifestyle, alcohol consumption, previous endemic infectious diseases, and reinfection were associated with the post-COVID-19 syndrome (*P* < 0.05). All these variables were added to the multivariate regression model. In the multivariate regression, female sex (incidence rate ratio [IRR]: 1.59; 95% CI: 1.44–1.76), non-White race (IRR: 1.14; 95% CI: 1.00–1.30), number of symptoms in the acute phase (IRR: 1.07; 95% CI: 1.06–1.09), body mass index (IRR: 1.01; 95% CI: 1.00–1.02), and reinfection (IRR: 1.46; 95% CI: 1.12–1.91) were independent predictors of increased risk of having persistent symptoms 12 months after an episode of COVID-19 (Table 2). In a sensitivity analysis, we verified whether the predictors differ according to race and found similar findings in both groups, suggesting that there was no modification of the effect by race (Supplemental Table 2). History of dengue, malaria, Zika, chikungunya, leprosy. and visceral leishmaniasis was not associated with late COVID-19 symptoms (IRR: 1.08; 95% CI: 0.96–1.22). In a sensitivity analysis after excluding those with less than 3 months between second infection episode and 12-month follow-up, similar findings were noted as in the primary analysis (Supplemental Table 3). Likewise, the results were similar using IPW (Supplemental Tables 4 and 5).

**Table 2:** Poisson regression of the number of late symptoms after 12 months of follow-up

Characteristics	Bivariate regression		Multivariable regression	
	IRR (95% CI)	*P* value	IRR (95% CI)	*P* value
Female, *n* (%)	1.75 (1.59–1.93)	< 0.001	1.60 (1.44–1.77)	< 0.001
Age, mean ± SD	1.00 (1.00–1.01)	0.20	–	–
Non-White race, *n* (%)	1.23 (1.08–1.40)	0.002	1.14 (1.00–1.30)	0.04
Comorbidities, mean ± SD	1.16 (1.08–1.24)	< 0.001	1.07 (0.99–1.14)	0.07
Hospitalization, *n* (%)	1.08 (0.90–1.29)	0.398	–	–
No. of symptoms in the acute phase, mean ± SD	1.09 (1.08–1.11)	< 0.001	1.07 (1.06–1.09)	< 0.001
BMI, mean ± SD	1.01 (1.01–1.02)	0.002	1.01 (1.00–1.02)	0.02
Sedentary lifestyle, *n* (%)	1.21 (1.11–1.33)	< 0.001	1.00 (0.91–1.11)	0.92
Smoking, *n* (%)	1.13 (0.93–1.38)	0.23	–	–
Alcoholism, *n* (%)	0.86 (0.75–0.98)	0.027	1.01 (0.87–1.16)	0.94
Reinfection, *n* (%)	1.35 (1.04–1.77)	0.024	1.45 (1.11–1.90)	0.006
Vaccine first or second dose, *n* (%)	1.00 (0.90–1.10)	0.92	–	–
Previous endemic infectious diseases, *n* (%)	1.15 (1.02–1.30)	0.017	1.07 (0.97–1.18)	0.18

BMI = body mass index; IRR = incidence rate ratio. BMI and race were absent for 10 and five participants, respectively.

## DISCUSSION

In a cohort of 1,371 patients followed for 12 months after the acute phase of COVID-19, 63.9% reported late COVID-19 symptoms. After adjusting for multiple confounders, female sex, non-White race, number of symptoms in the acute phase, body mass index, and reinfection were predictors of long-term symptoms. Previous endemic tropical diseases were not associated with long-term symptoms. The current study was performed in the Amazon Basin, a region with a high prevalence of tropical diseases. Although more than half of the individuals reported previous tropical infection, this characteristic was not associated with post-COVID-19 symptoms. Interestingly, Dengue accounts for 71% of all previous tropical disease cases and was associated with greater symptoms in the acute phase of COVID-19, but it was not associated with COVID-19 long-term symptoms.^7^

The most common late symptom was fatigue, affecting 43.5% of subjects. Recently published data from other studies in southeastern Brazil also point to fatigue as the most common late symptom. Other frequent symptoms, such as dyspnea headache and anosmia, are also similar to our findings.^11^^,^^12^ This high prevalence of fatigue may be associated with the various cognitive and neuropsychiatric symptoms that predict the occurrence of fatigue in the post-COVID-19 condition.^13^ The low mortality among men and women (0.60% and 0.53%, respectively), and the sample mostly represented with outpatient (93%), may have favored the association between female sex and a greater number of symptoms in the post-COVID-19 syndrome.^14^ Also, similar to other reports, the number of symptoms during the acute phase of COVID-19, female sex, and body mass index were predictors of late post-COVID-19 symptoms.^15^^,^^16^

In the current study, 32 participants (2.3%) had a second episode of SARS-CoV-2 infection, confirmed by RT-PCR, variants as Alpha, Beta, Gamma and Delta, known during the study period, may be responsible for reinfection. These persons were evaluated 2.3 months after the reinfection and were more likely to report persistent symptoms. After the sensitivity analysis, we found similar findings to those of the primary analysis, suggesting persistence of symptoms beyond the period of convalescence from the second episode (Supplemental Table 2). It is possible that reinfection enhances pathophysiological mechanisms involved in the development of the post-COVID-19 syndrome. Potential contributing factors to the development of post-COVID-19 syndrome include long-term tissue damage and pathological inflammation (e.g., viral persistence, immune dysregulation, and autoimmunity).^17^^,^^18^

Our study presented the prevalence of late symptoms of COVID-19 in an ethnically diverse population. We found that 64% of patients developed post-COVID-19 syndrome, which was relatively high compared with previous reports from Asia and Europe.^14^ The high proportion of non-White individuals (brown, black, and another race) in our study may partially explain these differences because non-White race was an independent predictor of late COVID-19 symptoms. No individual self-declared as Asian in our cohort.

This study has limitations. Because there is still complexity in the case definition of SARS-CoV-2 reinfection,^19^ our findings should be considered with caution. We used Poisson regression to count any symptom as an end point because post-COVID-19 syndrome lacks a strict definition.^20^ Although the questionnaire addresses the possible alternative diagnoses and patients were asked to report only *de novo* symptoms, we cannot assume that all self-reported late symptoms are related to COVID-19.

We also know that after acute infection some persons, especially certain immunocompromised individuals, continue to shed virus for long periods of time; in our cohort, we did not obtain genetic sequencing data of each presumed reinfection to excluded prolonged virus shedding. However, variants of concern such as Alpha, Beta, Gamma, and Delta, which known during the study period, may be responsible for reinfection.^21^ In addition, we used the CDC chronological criteria for reinfection considering that it is uncommon for the virus to persist for more 90 days after the initial infection.

The median time of 2.3 months between second infection and data collection is relatively short, and the convalescence (acute symptoms of reinfection) may play a role in the reported symptoms. We performed a sensitivity analysis after excluding those with a less than 3-month interval between reinfection and assessment in 12 months and analyzed 10 of the 32 individuals; there were similar findings in the association between reinfection and late symptoms. Whether these symptoms represented convalescence from the second episode or will persist for many months requires further follow-up to determine whether reinfection is an additional risk factor for prolonged persistence of symptoms post COVID-19. Recall bias is also possible because participants who reported reinfection may be more likely to recall post-COVID-19 symptoms than those without reinfection. Additionally, data on previous tropical infection were self-reported which may insert information bias. For instance, dengue has been reported to be asymptomatic in 30% of subjects.

In conclusion, in a population from the Amazon Basin, female sex, non-White race, number of symptoms in the acute phase, body mass index, and reinfection, but not previous endemic tropical diseases, were associated with long-term symptoms in a population with a high prevalence of symptoms in 12-months of follow-up.

## Supplemental Material


Supplemental materials
