# Supplementary material for: Prevalence and Predictors of COVID-19 Long-Term Symptoms: A Cohort Study from the Amazon Basin
Source: Am J Trop Med Hyg. 2023 Jun 26;109(2):466–70. doi: 10.4269/ajtmh.22-0362 (PMC10397456; doi:10.4269/ajtmh.22-0362)
Supplement: Supplementary file 1 [file tpmd220362.SD1.pdf]

Supplementary material (tables)

Supplemental Figure 1. Flowchart of subjects with COVID-19

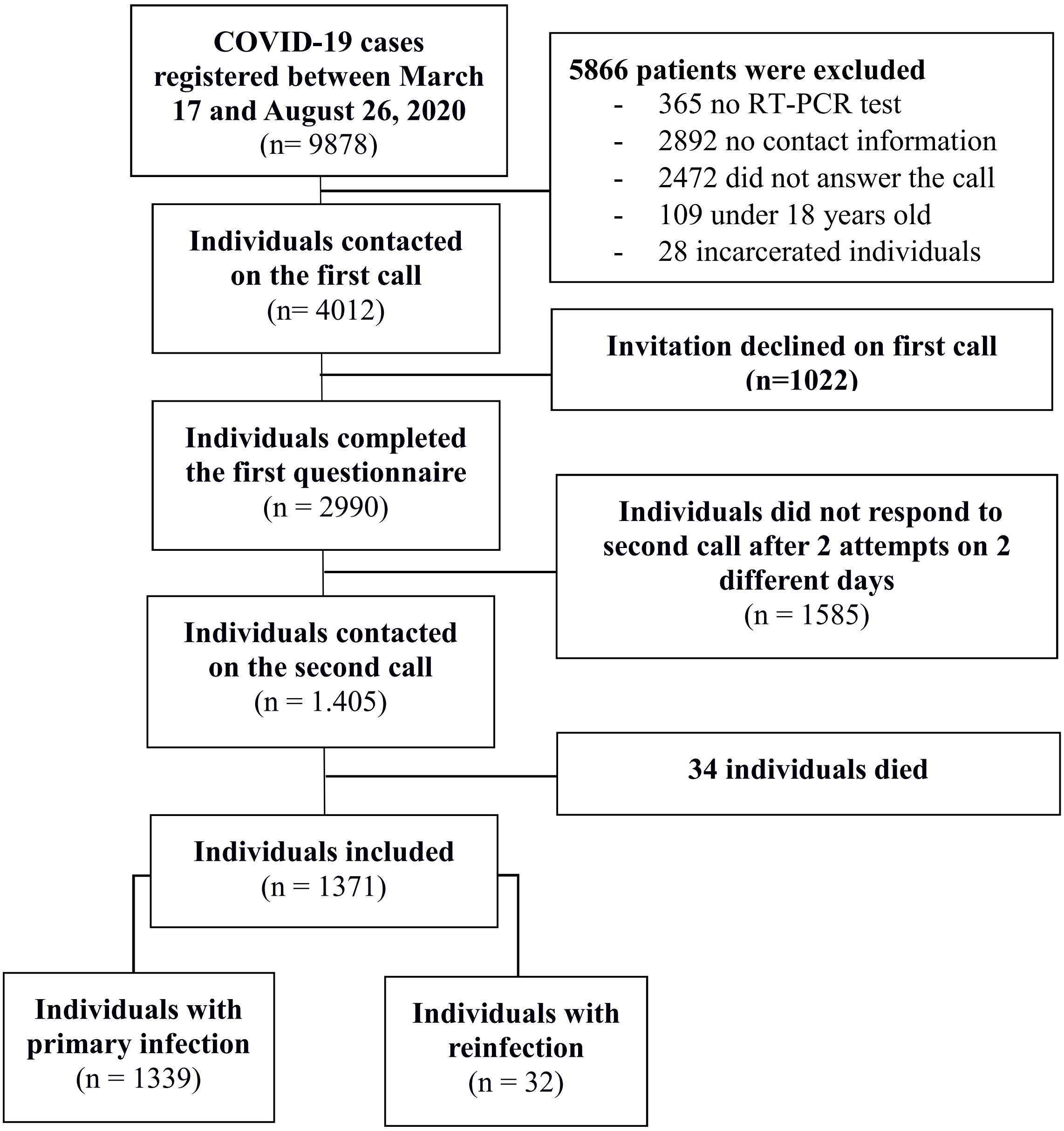

Supplemental Table 1. Symptoms reported after a median follow-up of 12 months

Symptoms > 12 weeks, n (%)	Total (n = 1371)	Primary infection (n = 1339)	Reinfection (n = 32)	P
Fatigue	597 (43.5)	578 (43.1)	19 (59.3)	0.06
Headache	219 (16.0)	211 (15.7)	8 (25.0)	0.15
Back pain	166 (12.1)	164 (12.2)	2 (6.2)	0.41
Dyspnea	120 (8.7)	116 (8.6)	4 (12.5)	0.51
Myalgia/Arthralgia	139 (10.1)	135 (10.0)	4 (12.5)	0.55
Chest pain	78 (5.7)	76 (5.6)	2 (6.2)	0.70
Anosmia	74 (5.4)	73 (5.4)	1 (3.1)	1.0
Palpitations	49 (3.5)	47 (3.5)	2 (6.2)	0.31
Ageusia	44 (3.2)	43 (3.2)	1 (3.1)	1.0
Dizziness	39 (2.8)	37 (2.7)	2 (6.2)	0.23
Cough	28 (2.0)	24 (1.8)	4 (12.5)	**0.003**
Sore throat	20 (1.4)	19 (1.4)	1 (3.1)	0.37
Abdominal pain	18 (1.3)	18 (1.3)	0 (0.0)	1.00
Coryza	18 (1.3)	18 (1.3)	0 (0.0)	1.00
Nasal congestion	11 (0.8)	10 (0.7)	1 (3.1)	0.23
Nausea	11 (0.8)	11 (0.8)	0 (0.0)	0.60
Anorexia	7 (0.5)	6(0.4)	1 (3.1)	0.15
Rash	5 (0.3)	5 (0.3)	0 (0.0)	1.00
Diarrhea	5 (034)	5 (0.3)	0 (0.0)	1.00
Fever	3 (0.2)	3 (0.2)	0 (0.0)	1.00
Odynophagia	3 (0.2)	2 (0.1)	1 (3.1)	0.06
Vomiting	2 (0.1)	2 (0.1)	0 (0.0)	1.00

Supplemental Table 2. Poisson regression among self-reported white individuals (n=251) and non-white individuals (n=1115).

	White		Non-White		
	**Multivariable regression**		**Multivariable regression**		**Interaction**
	**IRR (95% CI)**	**p**	**IRR (95% CI)**	**p**	**p**
Female	1.84 (1.41 – 2.39)	<0.001	1.56(1.39 – 1.74)	<0.001	0.28
Comorbidities	1.03 (0.87 – 1.22)	0.71	1.07 (0.99 – 1.16)	0.09	0.78
Number of acute symptoms	1.08 (1.04 – 1.13)	<0.001	1.07 (1.05 – 1.09)	<0.001	0.70
BMI	1.00 (0.98 – 1.02)	0.93	1.01 (1.00 – 1.02)	0.02	0.27
Sedentary lifestyle	0.90 (0.71 – 1.14)	0.39	1.03 (0.92 – 1.14)	0.65	0.39
Alcoholism	1.00 (0.71 – 1.41)	1.00	0.99 (0.86 – 1.17)	0.99	0.94
Reinfection	2.43 (1.12 – 5.29)	0.03	1.37 (1.03 – 1.82)	0.03	0.17
Previous endemic infectious diseases	1.00 (0.77 – 1.31)	0.96	1.08 (0.97 – 1.20)	0.15	0.61

BMI was absent for 10 participants.

Supplemental Table 3. Poisson regression of the number of late symptoms after 12 months of follow-up after excluding those with less than 3 months between second infection and data collection

	**Bivariate regression**		**Multivariable regression**	
	**IRR (95% CI)**	**p-value**	**IRR (95% CI)**	**p-value**
Female, n (%)	1.75 (1.59 – 1.93)	<0.001	1.59 (1.44 – 1.76)	<0.001
Age, mean ± SD	1.00 (1.00 - 1.01)	0.20		
Comorbidities, mean ± SD	1.16 (1.08 - 1.24)	<0.001	1.06 (1.00 - 1.14)	0.082
Hospitalization, n (%)	1.08 (0.90 – 1.29)	0.398		
Number of symptoms, mean ± SD	1.09 (1.08 -1.11)	<0.001	1.07 (1.06 - 1.09)	<0.001
BMI, mean ± SD	1.01 (1.01 - 1.02)	0.002	1.01 (1.00 – 1.02)	0.021
Sedentary lifestyle, n (%)	1.21 (1.11 - 1.33)	<0.001	1.01 (0.91 – 1.11)	0.86
Smoking, n (%)	1.13 (0.93 – 1.38)	0.23		
Alcoholism, n (%)	0.86 (0.75 – 0.98)	0.027	1.00 (0.87 – 1.55)	0.93
Reinfection, n (%)	1.55 (1.00 - 2.40)	0.052	1.57 (1.01 – 2.45)	0.043
Vaccine first or second dose	1.00 (0.90 - 1.10)	0.92		
Previous endemic infectious diseases	1.15 (1.02-1.30)	0.017	1.08 (0.95 -1.22)	0.20

IRR, relative risk; 95% CI, 95% confidence interval; SD, standard deviation; BMI, body mass index.

Supplemental Table 4. Baseline sociodemographic and clinical characteristics of patients evaluated 12 months after COVID-19 and patients not evaluated at 12-month follow-up.

**Characteristics**	**Total (n = 2944)**	**Individuals not evaluated after 12 months (n = 1573)**	**Individuals evaluated after 12 months (n = 1371)**	**p value**
Age (years), mean ± SD	40.1 ± 12.3	40.49 ± 12.9	39.6 ± 11.6	0.07
Female, n (%)	1518 (51.5)	823(52.3)	695 (50.6)	0.37
Smoking, n (%)	190 (6.4)	120 (7.6)	70 (5.1)	**0.005**
Alcohol, n (%)	414 (14.0)	203 (12.9)	211 (15.3)	0.05
Sedentary lifestyle, n (%)	1519 (51.6)	813 (51.6)	706 (51.5)	0.91
BMI, mean ± SD	28.4 ± 5.1	28.3 (5.1)	28.5 ± 5.2	0.45
Number of symptoms (acute phase), mean ± SD	8.7 ± 3.5	8.6 ± 3.6	8.8 ± 3.5	0.24
Previous endemic infectious diseases	1813(61.5)	956 (60.7)	857 (62.5)	0.33
Race, n (%)				0.08
White	550 (18.7)	299 (19.0)	251 (18.3)	
Brown	2056 (70.0)	1103 (70.2)	953 (69.7)	
Black	233 (7.9)	109 (6.9)	124 (9.0)	
Other	98 (3.3)	60 (3.8)	38 (2.7)	
Comorbidities, n (%)				
Hypertension	600 (20.3)	354 (21.9)	255 (18.6)	**0.02**
Chronic heart diseases	108 (3.6)	64 (4.0)	44 (3.2)	0.21
Diabetes mellitus	186 (6.3)	111 (7.0)	75 (5.4)	0.07
Chronic lung disease	123 (4.1)	66 (4.2)	57 (4.1)	0.95
Chronic kidney disease	41 (1.3)	19 (1.2)	22 (1.6)	0.35
Socioeconomic factors				
Education in years, mean ± SD	12.6 ± 4.1	12.2 ± 4.2	13.0 ± 3.9	**<0.001**
Monthly income in BRL Median (IQR)	2000 (1000-4000)	2000 (100-4000)	2000 (1000-4000)	**0.01**

Abbreviations: SD, standard deviation. BMI, Body Mass Index. IQR, interquartile range. BRL – Brazilian Real (1 BRL = 0.18 US Dollar)

BMI was missing for 41 participants; Race was missing for 7 participants; Education in years was missing for 8 participants; Monthly income in reais was missing for 480 participants.

Supplemental Table 5. Poisson regression of the number of late symptoms after 12 months of follow-up

	Bivariate regression		Multivariable regression	
	**IRR (95% CI)**	**p value**	**IRR (95% CI)**	**p value**
Female	1.75 (1.56 – 1.97)	<0.001	1.60 (1.42 – 1.80)	<0.001
Age in years	1.00 (0.99 – 1.00)	0.27		
Comorbidities	1.15 (1.06 – 1.24)	<0.001	1.05 (0.98 – 1.13)	0.14
Hospitalization	1.06 (0.84 – 1.32)	0.60		
Number of symptoms	1.09 (1.07 –1.10)	<0.001	1.07 (1.05 – 1.09)	<0.001
BMI	1.01 (1.00 – 1.02)	0.008	1.00 (1.00 – 1.01)	0.03
Sedentary lifestyle	1.20 (1.08 – 1.35)	0.001	1.00 (0.89 – 1.11)	0.97
Smoking	1.14 (0.89 – 1.46)	0.27		
Alcoholism	0.85 (0.73 – 0.99)	0.03	0.99 (0.85 – 1.14)	0.91
Reinfection, n (%)	1.38 (1.00 – 1.91)	0.04	1.51 (1.08 – 2.11)	0.01
Vaccine first or second dose	1.00 (0.88 – 1.12)	0.98		
Previous endemic infectious diseases	1.15 (1.03 –1.29)	0.01	1.06 (0.95 – 1.18)	0.24

IRR, relative risk; 95% CI, 95% confidence interval; SD, standard deviation; BMI, body mass index. BMI was missing for 12 participants.
